# Environmental and socio-demographic factors associated with cutaneous leishmaniasis in district Khyber, Pakistan; alarming spread of the disease to new foci

**DOI:** 10.1016/j.heliyon.2024.e29571

**Published:** 2024-04-16

**Authors:** Chao Lu, Zerman Ullah, Khurshaid Khan, Safeer Ullah Shah, Muhsin Jamal, Nazma Habib Khan

**Affiliations:** a Hefei University of Technology, Hefei, China; b Department of Zoology, Abdul Wali Khan University Mardan, Khyber Pakhtunkhwa, Pakistan; c National Centre of Excellence in Geology, University of Peshawar, Peshawar, Pakistan; d Department of Microbiology, Abdul Wali Khan University Mardan, Khyber Pakhtunkhwa, Pakistan; e Department of Zoology, University of Peshawar, Khyber Pakhtunkhwa, Pakistan

**Keywords:** Cutaneous leishmaniasis, Khyber, Spatial distribution, Risk factors, Pakistan

## Abstract

Cutaneous leishmaniasis (CL) is a sand fly-borne infection of significant public health concern in Pakistan (endemic for CL). This study aimed to scrutinize the environmental and socio-economic risk factors for CL in district Khyber (located on Pak-Afghan border), Pakistan. Clinically confirmed 2881 CL case records for (January–December) 2017 and 2020 were obtained from district hospital. In addition, a questionnaire for CL risk factors assessment was administered to 525 households (175 in 2017 and 350 in 2020) in 40 villages throughout the district in a household survey. Higher number of CL cases were recorded in 2020 (N = 1824 belonging to 90 villages) compared to 2017 (N = 1057 from 42 villages). Highest number of CL patient cases were recorded in tehsil Jamrud (N = 2248, 39.01 %), followed by Landi Kotal (N = 398, 6.91 %) and Bara (N = 235, 4.08 %). Records showed higher number of CL cases in males (N = 1,659, 57.58 %) compared to females (N = 1,222, 42.41 %). In 2017 and 2020 the disease burden peaked in January. GIS-based spatial analyses of hospital records revealed that CL cases were abundant at 294-1,916 m elevation, in agriculture and range lands. Univariate logistic regression model analysis of risk factors suggested that higher education, family size >15, knowledge of CL, having family member/s that suffered from CL in the past, knowledge about biting time of sand flies, use of mosquito spray, presence of Afghan refugees in the village/s and living in mud-made houses increased the risk of acquiring CL. The multivariable logistic regression model showed none of the risk factors to be statistically significant. Findings of the study are crucial towards effective and targeted control of CL in district Khyber.

## Highlights

• Scrutinized socio-economic and environmental risk factors associated with CL.
• Increased CL cases in 2020 compared to 2017 highlights a possible spread to new foci.
• GIS-based analyses showed that CL cases were abundant at 294-1,916 m elevation in Khyber.
• Findings of the study are imperative towards effective and targeted control of CL in the district.

## 1 Introduction

Leishmaniasis is caused by protozoan belonging to genus *Leishmania*. The disease is prevalent in more than 100 countries [1,2] afflicting approximately 1.5–2 million people annually [2]. Leishmaniasis has a diverse clinical spectrum ranging from self-healing cutaneous leishmaniasis (CL) to fatal systemic visceral leishmaniasis (VL) [3].The disease poses a significant burden on public health since it is still regarded as a neglected tropical disease [4]. Lack of any approved vaccine, urbanization, deforestation, human mass migration and climatic changes, have contributed towards the spread of the disease from endemic to new foci [5,6]. Spatial analysis using remote sensing (RS) and Geographic Information System (GIS) with ecological and climatic factors are operational tools for understanding high risk transmission spots, forecasting disease control plans, modelling disease transmission and predicting future outbreaks [1,7,8]. Similarly, identifying risk factors for leishmaniasis is critical for designing community-level responses toward control of the disease [1,[8], [9], [10]].

In Pakistan, both CL and VL forms of the disease are prevalent however, CL cases are relatively more frequent [1,11]. CL is particularly endemic in regions throughout the southwestern province of Baluchistan and northwestern province of Khyber Pakhtunkhwa (KP) [1,3]. *Leishmania tropica*, *L. major,* and rarely, *L. infantum* are responsible for causing CL in the country [1,12,13]. Reports indicate CL as a *trans*-boundary disease between Pakistan and Afghanistan [[14], [15], [16], [17], [18]]. Afghanistan has one of the highest burdens of CL [18,19] and has been on a rise due to political instability, war conflicts and frequent human displacement [20]. Regular movement of migrants, refugees and traders are some of the major factors responsible for the spread of the disease to non-endemic foci in bordering areas of Pakistan particularly the merged tribal districts including Khyber (formerly Khyber Agency of FATA, Federally Administrated Tribal Areas) [1,8,16]. Primary strategic significance of Khyber is the Khyber Pass which is a major route for trading and mass migration of Afghans. Outbreaks of CL have been reported in the district by local media, national and international health organizations [[21], [22], [23], [24]]. *L. tropica* causing ACL has been reported from foci of Khyber [25].

Access to proper healthcare remains huge obstacle for leishmaniasis control in Pakistan, especially for rural communities, informal settlements and areas affected by conflict such as district Khyber. The present study aimed to investigate the environmental and socio-demographic risk factors for CL in the district of Khyber, Pakistan. The region has been poorly explored for CL, its reservoirs and vectors due to historical conflict and poor federal legislation [[26], [27], [28]].

## 2 Methodology

### 2.1 Study area

**Fig. 1:**
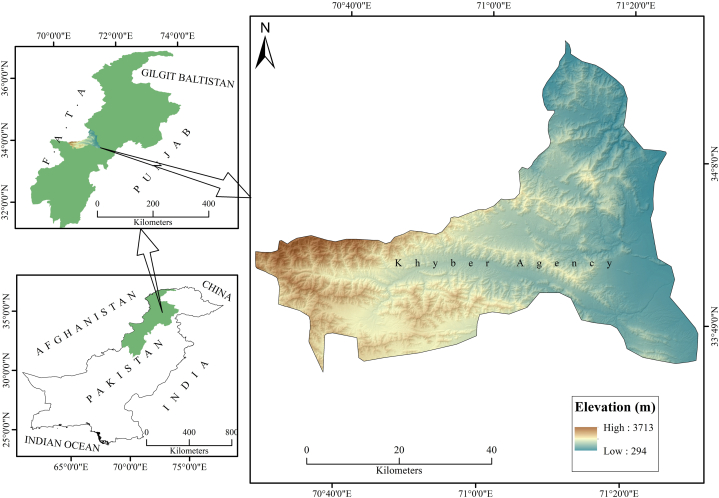
Study area map of district Khyber.

The study was conducted in District Khyber (33^0^ N and 71^0^ E), Khyber Pakhtunkhwa (KP), Pakistan. The district comprises four tehsils (administrative sub-unit of the district) Bara, Jamrud, Landi Kotal, and Mula Gori with a total surface area of 2576 Km^2^. The district shares its North with River Kabul (originating from Afghanistan) and Koh-e-Suffaid range, South with district Orakzai, East with district Peshawar, West with district Kurram, Northwest with Afghanistan, and Northeast with district Mohmand (Fig. 1). The district topography varies from plain to small elevated mountains.

### 2.2 Study design

The focus of this study was two-fold. Firstly, the study retrospectively assesses seasonal and spatial CL pattern based on hospital records obtained for district Khyber. Secondly, a cross-sectional household survey was conducted to assess prevalent risk factors for the disease based on in-person questionnaires.

### 2.3 Archived CL data from hospital

Clinically confirmed CL patient data for years 2017 and 2020 (January–December) was collected from the registry of Civil Hospital Jamrud (Leishmaniasis Centre, providing free of cost treatment) district Khyber. The registry record included patient name, gender, age, date of visit, village, site of infection etc. Mean monthly data for rainfall and temperature in 2017 and 2020 was acquired from Meteorological station in Peshawar, Khyber Pakhtunkhwa.

### 2.4 CL risk factors assessment

This survey was conducted in three tehsils (Jamrud, Landi Kotal, and Bara) of district Khyber. For assessment of behavioral and demographic CL risk factors, a questionnaire was administered to household heads of randomly selected 525 households (175 in 2017 and 350 in 2020) in 40 villages throughout the district. Informed written consent (signed or thumb printed) was obtained before inclusion of households in the study. This study was approved by Ethics Research Committee Abdul Wali Khan University Mardan (reference no. ZOO-AWKUM/16003294/2017). Approval was also obtained for acquiring CL records from the Civil Hospital Jamrud administration (reference no. DHQ-LC/KH/2017&2020).

### 2.5 Statistical analysis

CL records, risk factors and acquired climatic data and co-ordinates were transferred to Microsoft Excel for further statistical analysis.

Global positioning system (GPS) co-ordinates data for the locality of each patient record from archived data was acquired using Google EarthPro and GPS device. The co-ordinate's data was then shifted to ArcGIS (version 10.5), Environmental System Research Institute (ESRI) USA, for spatial mapping. A Digital Elevation Modal (DEM computer based, freely online accessible elevation data launched by NASA-USA and MITI-Japan in 1999) of the district was acquired from Advance Spaceborne Thermal Emission and Reﬂection Radiometer (ASTER) to correlate the possible association between CL cases and elevation (m). Briefly, we acquired a 12.5 m resolution ALOS (Advanced Land Observing Satellite) PALSAR (Phased Array type L-band Synthetic Aperture Radar) digital elevation model for the study area. Land-use/land-cover projection maps of the district were obtained from the National Centre of Excellence in Geology, University of Peshawar, KP, Pakistan. Land-use maps had pre-described classifications of land-cover and water bodies (streams, lacks and reservoirs) which were used for regional measurement of land-use and land-cover data of the study area (USGS, United States geological survey).

Risk factors for CL in the district were assessed using univariable and step-wise multivariable regression model using Stata Corp LP, 2013. The outcome for risk factors analysis was the presence of CL history within the household based on question “Did any of your family member suffer from CL in the past one year?”

## 3 Results

### 3.1 CL patient records from district Khyber

A total of 2881 clinically confirmed CL patient's data (2017 and 2020) was acquired from the district hospital. Higher number of CL cases were recorded in 2020 (N = 1,824, 63.31 %) as compared to 2017(N = 1,057, 36.68 %). Overall, CL infection was higher in males (N = 1659) compared to females (N = 1222).CL infection was common in age group <26 years. Majority of the patient's presented lesions on their face (Table 1).

**Table 1:** Clinico-demographic data of cutaneous Leishmaniasis patient's from Khyber (2017 and 2020).

2017				2020			
**Gender**	**Number**	**Percentage**	**95 % confidence interval**	**Gender**	**Number**	**Percentage**	**95 % confidence interval**
Female	423	40.02	0.37─0.43	Female	846	53.62	0.44─0.49
Male	634	59.98	0.57─0.63	Male	978	46.38	0.51─0.56
**Age**							
<26	898	84.96	0.83─0.87	<26	1541	84.48	0.83─0.86
26–35	58	5.49	0.04─0.07	26–35	100	5.48	0.05─0.07
>35	101	9.56	0.08─0.11	>35	183	10.03	0.09─0.11
**Site of lesion/infection**							
Face	432	40.87	0.38─0.44	Face	647	35.47	0.33─0.38
Leg/s	259	24.50	0.22─0.27	Leg/s	391	21.44	0.20─0.23
Arm/s	295	27.91	0.25─0.31	Arm/s	592	32.46	0.30─0.35
Mixed	71	6.72	0.05─0.08	Mixed	194	10.64	0.09─0.12

CL patients visiting the health facility belonged to 132 villages (42 in 2017 and 90 in 2020) of three tehsils (Supp Table 1). Maximum number of CL patient cases were recorded in tehsil Jamrud (N = 2,248, 78.03 %), followed by Landi Kotal (N = 398, 13.81 %) and Bara (N = 235, 8.16). (Table 2).

**Table 2:** Tehsil-wise distribution of cutaneous Leishmaniasis in District Khyber (2017 and 2020).

Year	Villages	Gender	Jamrud	Landi Kotal	Bara	Grand total
2017	42	Male	412 (62.61)	141 (53.81)	81 (59.12)	634
		Female	246 (37.38)	121 (46.18)	56 (40.87)	423
		Total	658	262	137	1057
2020	90	Male	891 (56.03)	69 (50.73)	65 (66.32)	1025
		Female	699 (43.96)	67 (59.26)	33 (33.67)	799
		Total	1590	136	98	1824
	132	Total	2248 (78.03)	398 (13.81)	235 (8.16)	2881

**Fig. 2:**
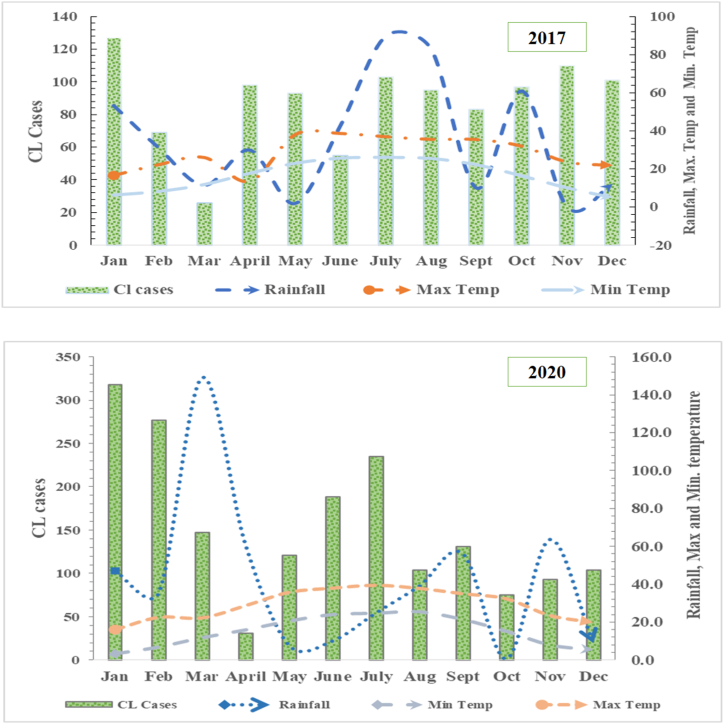
Prevalence of cutaneous leishmaniasis in Khyber.

In both 2017 and 2020, CL cases peaked in January where minimum values of monthly rainfall and temperature were observed (Fig. 2). However, monthly patient burden in did not correlate significantly (p-value>0.05) with mean monthly rainfall and temperature (Supp Table 2).

**Fig. 3:**
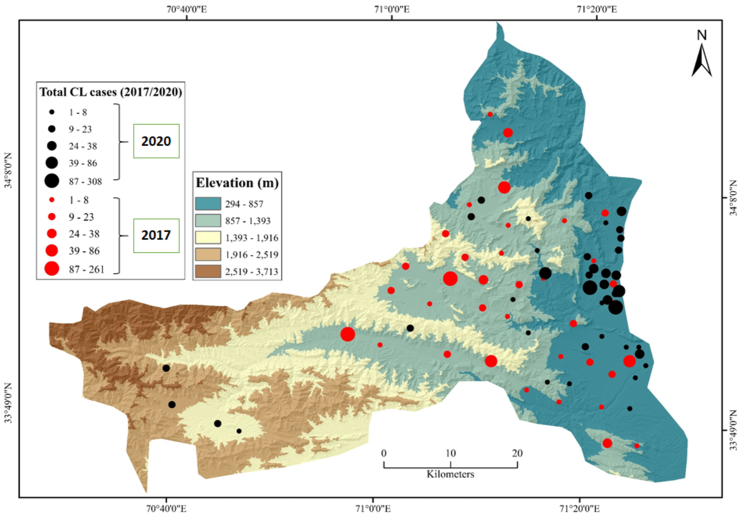
Cutaneous leishmaniasis projected on DEM.

**Fig. 4:**
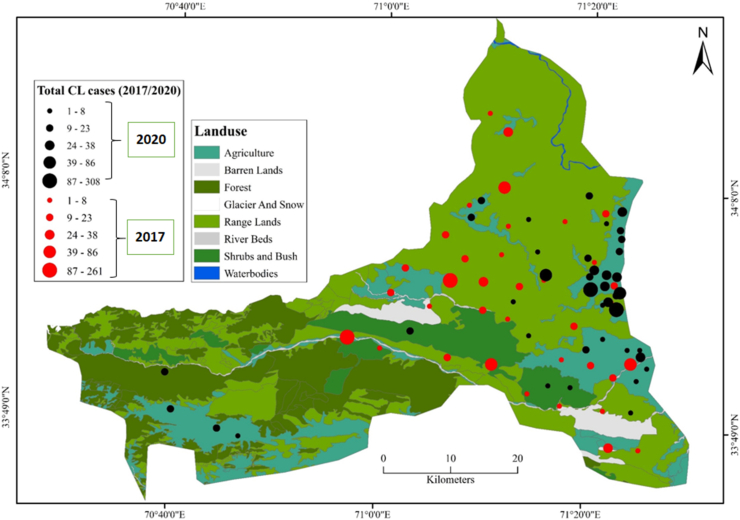
Cutaneous leishmaniasis projected on land cover map.

Spatial analysis performed for CL case records from the district revealed that majority of the cases originated from regions at 294-1,916 m elevation (Fig. 3). In 2020, CL cases seemed to be more clustered compared to 2017, since the patients in 2020 belonged to villages that were in close proximity to each other. CL cases were observed in maximum numbers on the agriculture and range lands of the district (Fig. 4).

### 3.2 Risk factors for CL

**Fig. 5:**
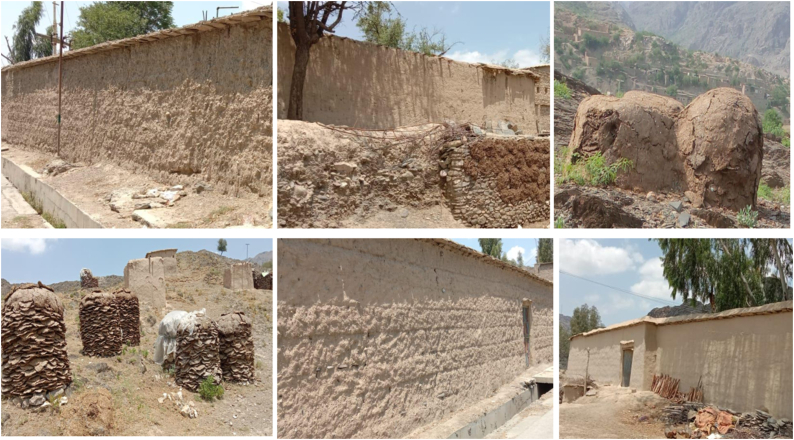
Mud houses with breeding sites for sand flies.

Univariable analysis performed on risk factors data acquired from 525 households of Khyber indicated that respondent's education (OR = 2.82; 95 % CI = 1.67─4.74), having a family size >15 (OR = 2.07; 95 % CI = 1.20─3.56), knowledge of CL (OR = 1.93; 95 % CI = 1.32─2.82), presence of family member/s that suffered from CL in household (OR = 0.02; 95 % CI = 0.01─0.05), knowledge about biting time of sand flies (OR = 1.84; 95 % CI = 1.24─2.71), use of insecticides (OR = 1.81; 95 % CI = 1.44─2.87), Afghan refugees living in the village/s (OR = 3.24; 95 % CI = 2.17─4.84) and living in mud-made house (OR = 1.88; 95 % CI = 1.27─2.78) (Fig. 5) increased the risk of acquiring CL, (p-value <0.01). In multivariable logistic regression, none of the risk factors proved statistically significant (Table 3).

**Table 3:** Univariable and multivariable analysis of CL risk factors.

Risk variables	Number	Percentage	Univariable analysis		Multivariable analysis	
			Odds ratio (95 % confidence interval)	p-value	Odds ratio (95 % confidence interval)	p-value
**Tehsil**						
Bara	101	19.24 %				
Landi Kotal	144	27.43 %	1.00			
Jamrud	280	53.33 %	0.79(0.53─1.19)	0.27		
**Age**						
<26	256	48.76 %	1.00			
26–35	172	32.76 %	1.23(0.81─1.86)	0.33		
>35	97	18.48 %	1.30(0.79─2.14)	0.30		
**Education**						
Primary	252	48.00 %	1.00			
Secondary	193	36.76 %	1.30(0.86─1.97)	0.21		
Higher	80	15.24 %	2.82(1.67─4.74)	<0.01	0.13(0.01─1.25)	0.08
**Nationality**						
Pakistani	521	99.24 %	1.00			
Other	4	0.76 %	6.53(0.67─63.26)	0.11		
**Occupation**						
Teacher	108	20.57 %	1.00			
Farmer	270	51.43 %	0.78(0.48─1.27)	0.32		
Businessman	106	20.19 %	1.12(0.64─1.96)	0.70		
Medical person	41	7.81 %	1.42(0.68─2.97)	0.36		
**Family-sized**						
<7	96	18.29 %	1.00			
7–15	221	42.10 %	1.24(0.71─2.15)	0.45	1.53(0.14─16.35)	0.73
>15	208	39.62 %	2.07(1.20─3.56)	<0.01	10.54(0.92─121.25)	0.06
**Socio-economic status**						
Low	187	35.62 %	1.00			
Middle	332	63.24 %	1.39(0.94─2.07)	0.10		
Elite	6	1.14 %	1.33(0.24─7.50)	0.74		
**Vegetation**						
No	280	53.33 %	1.00			
Yes	245	46.67 %	0.81(0.56─1.17)	0.27		
**Children are given fully covered with clothes**						
No	505	96.19 %	1.00			
Yes	20	3.81 %	0.52(0.17─1.59)	0.26		
**Knowledge of CL**						
No	338	64.38 %	1.00			
Yes	187	35.62 %	1.93(1.32─2.82)	<0.01	0.84(0.15─4.69)	0.84
**Family member/s suffered from CL**						
No	88	16.76 %	1.00			
Yes	437	83.24 %	0.02(0.01─0.05)	<0.01	1.68(0.12─24.35)	0.70
**Presence of mosquito bed net in the house**						
No	136	25.90 %	1.00			
Yes	389	74.10 %	1.22(0.80─1.87)	0.36		
**Knowledge of sand flies**						
No	518	98.67 %	1.00			
Yes	7	1.33 %	1.62(0.36─7.32)	0.53		
**Can differentiate between sand flies and mosquito**						
No	515	98.10 %	1.00			
Yes	10	1.90 %	0.92(0.23─3.59)	0.90		
**Knowledge about biting time of sand flies**						
No	211	40.19 %	1.00			
Yes	314	59.81 %	1.84(1.24─2.71)	<0.01	3.80(0.69─20.86)	0.12
**Use of mosquito spray**						
No	432	82.29 %	1.00			
Yes	93	17.71 %	1.81(1.14─2.87)	<0.01		
**Afghan refugees living in the village/s**						
No	240	45.71 %	1.00			
Yes	285	54.29 %	3.24(2.17─4.84)	<0.01	0.17(0.02─1.84)	0.15
**Do preachers visit the village**						
No	73	13.90 %	1.00			
Yes	452	86.10 %	1.96(1.07─3.57)	0.03		
**Type of house**						
Mixed	372	70.86 %	1.00			
Mud-made	153	29.14 %	1.88(1.27─2.78)	<0.01	1.49(0.30─7.34)	0.62
**Presence of domestic animals**						
No	100	19.05 %	1.00			
Yes	425	80.95 %	1.41(0.87─2.31)	0.17		
**Presence of cattle corrals**						
No	178	33.90 %	1.00			
Yes	347	66.10 %	0.72(0.49─1.06)	0.10		
**Presence of domestic chickens**						
No	53	10.10 %	1.00			
Yes	472	89.90 %	1.09(0.59─2.02)	0.79		
**Status of the house**						
Single story	434	82.67 %	1.00			
Double story	82	15.62 %	1.02(0.61─1.69)	0.95		
Other	9	1.71 %	2.74(0.72─10.36)	0.14		

## 4 Discussion

Leishmaniasis is currently extending its horizons from tropical to sub-tropical regions of the world [29]. It is a major public health issue, mainly affecting poverty ridden communities with limited health facilities [30]. Rapid urbanization, migration, civil wars, and nutritional standards are some of the major factors that are known to play essential roles in the global spread of leishmaniasis [31]. In Pakistan, CL is endemic in Baluchistan and became highly prevalent in various parts of KP (including the present study area of district Khyber) [12]. The disease is reported throughout the year with frequent outbreaks and an alarming geographical spread in the last few decades. Previous studies reported *L. tropica* causing ACL (anthroponotic cutaneous leishmaniasis) in the region and recently *P. sergenti* the suspected vector of the disease has also been identified in various parts of the district [3,25,32].

Our findings of confirmed CL cases revealed an expansion of CL in the district. Trends in reported CL cases during 2017 and 2020 indicated a spread of CL to non-endemic sites (villages). However, this study was based on registered statistics and therefore may not depict a true figure for burden of CL since is self-healing and many effected individuals usually self-medicate at homes [33]. We recorded highest prevalence in Jamrud tehsil of the district probably because this tehsil is the most populated with clustered human settings compared to other tehsils. Living in dense human settlements has been suggested as a significant factor for anthroponotic transmission of leishmaniasis [9,34,35].

In 2017 and 2020 records, maximum CL cases were reported in January. There was no linear trend of increase or decrease observed in other months, which may signify the disease's transmission and incubation period [36]. Climatic factors (temperature and rainfall) may contribute to the spread of CL by indirectly influencing sand fly life cycles, reservoir abundance, disease transmission pattern, and geographic scope of pathogens [34]. In Pakistan, sand flies are known to show an uni-model (single peak per year) distribution and are abundant from June to September [1,34,37]. Transmission of CL followed by an incubation period (5–6 months) correspond with the higher influx of CL cases from January to March in the health units in KP [27,38]. Although impact of COVID-19 on CL management cannot be ignored. A spike in CL cases during and after the pandemic highlight the negligence of Government health organizations due to shift in national focus towards controlling COVID-19 [[39], [40], [41]].

Hospital records from Khyber revealed that the infection was higher in males than females probably because vocational activities of males expose them to potential sand fly bites [8]. For instance, Males usually have outdoors activities and sleep in open places during summer which may increase their risk of sand fly bites [42,43]. We witnessed that maximum CL patients in hospital records were <26 years of age. Age plays a vital role in spreading and acquiring any disease. It has been reported that CL can affect all age groups however, younger age groups are more prone to acquiring the disease probably due to poor immunity in hyper-endemic situations [44]. We observed that many patients in district Khyber had lesions on their exposed parts, particularly on their face probably because locals do not cover their faces during active hours of sand flies [10,45] and to a greater extent on their vocational and shelter conditions [8,16,26,27,46].Sand flies activities have been observed to peak on warm and clear nights (indoor and outdoor) with low wind speed [8,47].

In the current study, we spatially analysed CL cases reported by district hospital of Khyber. The CL patients commonly originated from areas with elevation 294-1,393 m however, maximum number of cases were observed at 294-1,916 m in the district [1,8,12]. In Pakistan, the disease has been predominantly observed at an elevation ranging from 294 to 800 m in the country [1,48,49]. Elevation has been suggested as a significant environmental factor since suspected vector species for ACL (*P. sergenti*) has been reported to inhabit these high altitudes [34,49]. We also recorded higher CL burden on sites with agricultural lands, range land, forest, shrub and bushes. Living in proximity to forested areas, range lands and vegetation in general are known to increase risk of acquiring CL [50]. In Pakistan, studies reported that the suspected vector sand fly species (*P. sergenti* and *P. papatasi*) of CL are environmental generalists and can be found in a diverse range of ecological setups [48].

We demonstrated that the incidence of cutaneous leishmaniasis is influenced by a variety of socio-demographic and behavioral risk factors. We identified family size per room/dwelling as a risk factor for CL in the district. Higher number of individuals per dwelling reflect low socio-economic status Studies within the province have reported that congested housing foster higher risk of disease transmission among family members [8,51,52]. We observed that literacy and knowledge of CL was not protective against acquiring CL. One reason could be that despite the disease knowledge, locals could not adopt or afford to adopt protective measures [37,48]. Having Afghan refugees in neighborhood increased CL risk in Khyber. The current study area of district Khyber serves as a main route (Khyber Pass) for frequent trading and *trans*-boundary movement with connecting with Afghanistan [12,[14], [15], [16], [17], [18]]. Afghans are susceptible to acquiring the disease since they travel frequently between endemic areas of Afghanistan and Pakistan. We identified mud-made house construction to elevate the risk of CL. Locals here commonly use cattle dung mixed with mud for cementing mud walls, thus providing humid sites for sand fly oviposition and shelter in transmission season [35,53]. Step-wise multivariate analysis remained insignificant for any risk factor probably because stepwise methods are sensitive to the sample size, the order of the variables, corelation among variables and the significance level [54].

Due to financial constraints, security issues arising due to merging of the region with KP province and COVID-19, the study was carried out in two stages with a gap of two years.

## 5 Conclusion

It is evident from the study findings that CL is a serious public health concern in the district of Khyber, Pakistan. The rise in recorded cases in 2020 compared to 2017 may highlight a probable extent of CL to non-endemic sites.

Environmental (elevation and land-use) and climatic factors (temperature and rainfall) have been suggested to increase in CL incidence in the area. The present investigation also recognized certain that poor living conditions was significantly contributing towards the existing disease burden. We therefore, emphasize the region's dire need for vector control and disease awareness programs.

## Data availability

Data included in article/sup. materials/in referenced article.

## Ethics statement

The study was approved by board of studies ethics committee (reference no. ZOO-AWKUM/16003294/2017), Abdul Wali Khan University Mardan, Khyber Pakhtunkhwa, Pakistan.

## CRediT authorship contribution statement

**Chao Lu:** Resources, Funding acquisition. **Zerman Ullah:** Investigation, Data curation. **Khurshaid Khan:** Writing – original draft, Validation, Supervision, Investigation, Formal analysis, Conceptualization. **Safeer Ullah Shah:** Visualization, Software, Formal analysis. **Muhsin Jamal:** Software, Project administration, Formal analysis. **Nazma Habib Khan:** Writing – review & editing, Writing – original draft, Validation, Resources, Methodology, Investigation, Conceptualization.

## Declaration of competing interest

The authors declare that they have no known competing financial interests or personal relationships that could have appeared to influence the work reported in this paper.
