# Supplementary material for: Environmental and socio-demographic factors associated with cutaneous leishmaniasis in district Khyber, Pakistan; alarming spread of the disease to new foci
Source: Heliyon. 2024 Apr 16;10(8):e29571. doi: 10.1016/j.heliyon.2024.e29571 (PMC11053183; doi:10.1016/j.heliyon.2024.e29571)
Supplement: Multimedia component 2 [file mmc2.docx]

**Supp Table 2. Correlation of Cutaneous leishmaniasis cases (2017 and 2020) with climatic factors.**

| Year | **Rainfall (mean)** | **Mean maximum temperature** | **Mean minimum temperature** |
| --- | --- | --- | --- |
| 2017 | 0.24 | -0.25 | -0.14 |
| 2020 | -0.03 | -0.24 | -0.25 |
